# IL-33/ST2 Activation Is involved in Ro60-Regulated Photosensitivity in Cutaneous Lupus Erythematosus

**DOI:** 10.1155/2022/4955761

**Published:** 2022-07-20

**Authors:** Yitian Song, Fangzhi Wei, Ying Liu, Feng Han, Lihui Ma, Yanping Zhuang, Chengdan Pan, Zhandong Jia, Aimin Gong

**Affiliations:** ^1^Department of Internal Medicine of Traditional Chinese Medicine, Hainan Medical University, Haikou, 571199 Hainan, China; ^2^Department of Rheumatology and Immunology, Hainan General Hospital, Haikou, 570311 Hainan, China; ^3^Department of Laboratory Medicine, The First Affiliated Hospital of Hainan Medical College, Haikou, 570102 Hainan, China; ^4^Department of Rheumatology and Immunology, The First Affiliated Hospital of Hainan Medical College, Haikou, 570102 Hainan, China; ^5^Department of Nephrology, Ningbo Hospital of Traditional Chinese Medicine, Affiliated to Zhejiang Chinese Medical University, Ningbo, 315000 Zhejiang, China

## Abstract

Interleukin- (IL-) 33 contributes to various inflammatory processes. IL-33/ST2 activation participates in systemic lupus erythematous via binding to the receptor of Suppression of Tumorigenicity 2 protein (ST2). However, whether IL-33/ST2 interferes with the nosogenesis of cutaneous lupus erythematosus (CLE) has not been reported so far. Herein, we proposed to disclose the impacts on IL-33/ST2 activation and Ro60 on CLE and their potential implications in the photosensitization of CLE cells. IL-33, ST2, and Ro60 in CLE patients' skin lesions were detected. Murine keratinocytes stimulated with or without IL-33 were irradiated by ultraviolet B (UVB), and the levels of Ro60 and inflammation markers were determined. Keratinocytes were cocultured with J774.2 macrophages and stimulated with IL-33 for analysis of chemostasis. The results identified that IL-33, ST2, and downstream inflammation markers were significantly upregulated in CLE lesions with Ro60 overexpression. Additionally, IL-33 treatment promoted the upregulation of Ro60 induced by UVB treatment in murine keratinocytes. Moreover, IL-33 stimulates keratinocytes to induce macrophage migration via enhancing the generation of the chemokine (C–C motif) ligands 17 and 22. Meanwhile, the silencing of ST2 or nuclear factor-kappa B (NF-*κ*B) suppression abolished IL-33-induced upregulation of Ro60 in keratinocytes. Similarly, the inhibition of SOX17 expression was followed by downregulation of Ro60 in keratinocytes following IL-33 stimulation. In addition, UVB irradiation upregulated SOX17 in keratinocytes. Conclusively, the IL-33/ST2 axis interferes with Ro60-regulated photosensitization via activating the NF-*κ*B- and PI3K/Akt- and SOX17-related pathways.

## 1. Introduction

Cutaneous lupus erythematosus (CSF), which is found in 72-85% of lupus erythematosus (LE) cases, is a characteristic symptom of the disease in 23-28% of patients [1–3]. Commonly occurring in skin exposed to sunlight, the hallmarks of CLE are epidermal atrophy, keratinocyte (KC) apoptosis, and inflammatory cell infiltration due to the build-up of proteins involved in inflammation [4–8]. As an indicator of SLE activity and progression, there is a need to elucidate the pathogenetic mechanisms underlying inflammation in SLE, which could help monitor and contain the progression of SLE.

One of the major processes of CLE is the photosensitization of KCs during disease initiation and progression to the acute stage [4–8]. In CLE, photosensitivity results from ultraviolet irradiation involving the regulation of a number of genes [9–11]. Studies have shown, for example, that the expression of the Ro52 protein in skin KCs is upregulated by UV radiation, which leads to the release of autoantibodies against Ro52 [7]. Ro60 is an autoantigen that can bind to RNAs; studies have detected the presence of Ro60-specific antibodies in autoimmune diseases such as SLE, neonatal lupus, or Gougerot-Sjögren syndrome [12–15]. Ro60 plays a regulatory role in interferon- (IFN-) regulated local inflammation and immune response [16, 17]. The accumulation of antibodies against Ro60 in the skin of CLE has also been reported [14, 18]. Therefore, photosensitization regulated by Ro60 is central in the nosogenesis of CLE.

Interleukin- (IL-) 33 is a regulator of inflammatory processes. IL-33 activates its Suppression of Tumorigenic Protein 2 (ST2) receptor, thereby regulating various types of cells under inflammatory conditions [19, 20]. The latest evidence has shown the role played by IL-33/ST2 in fibrotic disorders, including cardiac, hepatic, pancreatic, cutaneous, cystic, and pulmonary fibrosis [21–23]. Mok et al. revealed no evident change in serum IL-33 between the control group and SLE patients but showed significantly elevated ST2, a marker that was linked to SLE disease activity and severity, demonstrating that ST2 may be a surrogate marker of disease activity [24]. The IL-33/ST2 interaction has been shown to interfere with the nosogenesis of systemic LE including lupus nephritis and other human diseases [25–28]. Similar to IL-1*β*, extracellular IL-33 activates intracellular signaling pathways via binding to ST2, a cell surface receptor [29], while in KCs, ST2 expression is stimulated by ultraviolet B (UVB) rays [25–28]. Thus, IL-33/ST2 axis activation is suggested to participate in the nosogenesis of CLE.

Considering that both ST2 and Ro60 levels are increased by UVB in KCs [29–33], we hypothesized the presence of a regulatory relationship between the IL-33/ST2 axis and Ro60 in CLE that might play a significant role in CLE, which has yet to be elucidated. Therefore, the novelty and purpose of this study are to clarify the possible role played by the IL-33/ST2 axis in photo-provoked injury in CLE and investigate the relationship between the IL-33/ST2 axis and Ro60 in CLE for the first time.

## 2. Data and Methods

### 2.1. Source of Tissue Specimens

Lesional (*n* = 26) and nonlesional cutaneous (*n* = 18) biopsies were obtained from treatment-naive patients diagnosed with CLE [34]. Control cutaneous tissue specimens (*n* = 15) were collected from healthy individuals during esthetic operations. The Hospital Research Ethics Committee approved this research that was carried out following the Declaration of Helsinki, and informed consent was obtained from all participants.

### 2.2. Immunofluorescence (IF) studies

The tissues were fixed in formalin and embedded in paraffin for sectioning. Paraffin sections were then deparaffinized and rehydrated and the subsequently treated with dual endogenous enzyme block that was ordered from DAKO, Glostrup, Denmark. The tissues and cells were cultivated overnight with either rabbit anti-ST2, SOX17, or rabbit anti-IL-33 (Abcam, Cambridge, MA, USA, cat. nos. ab284649, ab224637, and ab187060), and anti-Ro60 (cat. no.) IgG diluted to 1 : 100 served as the *Ι* antibody (4 *μ*g/ml, 4°C). Subsequently, phosphate-buffered saline (PBS) was used for washing, and Alexa Fluor 594- (red) and 488- (green) conjugated II antibodies (1 : 1000) supplied by Invitrogen, Grand Island, NY, USA, were utilized for incubation (1 h, 37°C). Then, the nucleus staining was conducted in a mounting medium added with DAPI (4,6-diamidino-2-phenylindole). Isotype IgG antibodies of each fluorochrome were used as nucleic controls. Image acquisition was conducted with the use of a Leica DFC300 FX fluorescent microscope. The software ImageJ1.61u supplied by National Institutes of Health, Bethesda, MD, USA, was used for the determination of positively stained areas. Four to six viewing fields were chosen randomly within each section. Three blinded pathologists performed the analysis, and the percentage of positive staining areas was calculated.

### 2.3. Cell Culture

PAM212 cells (Lonza Walkersville, Inc.) were seeded in a medium composed of Eagle's minimal essential media, 10% FBS, l-glutamine, 4-(2-hydroxyethyl)-1-piperazineethanesulfonic acid buffer, sodium pyruvate, nonessential amino acids, and penicillin-streptomycin (Gibco, Waltham, MA, USA). First, cells were treated with 24 h of starvation in a 2% FBS-added medium. Then, UVB irradiation (5.5 mJ/cm^2^) was performed with the use of an UVB device (302 nm) supplied by Rayminder, Ottawa Hills, OH, USA. Following this, cells in the stimulation groups were given 100 ng/ml recombinant IL-33 (ACROBiosystems) or 100 ng/ml bovine serum albumin (Gibco, USA) intervention. After 2 h of NF-*κ*B inhibitor JSH-23 (purity ≥ 98%, 20 *μ*M; Cayman), wortmannin (WM; 25 *μ*g/ml; Sigma), and U0126 (10 *μ*M; Promega) intervention, UVB and IL-33 interventions were carried out.

### 2.4. qRT-PCR

Total RNA was obtained from cells or tissues via extraction with a TRIZOL RNA isolation kit (Invitrogen). This was followed by reverse transcription into cDNA using a cDNA kit (TaKaRa, Japan). The qRT-PCR was then run on the CFX Opus 96 Real-Time PCR System, and the fluorescent dye used SYBR Green Master Mixes (Invitrogen). The 2^−ΔCt^ method calculated gene expression relative to *β*-actin. Sequences of the primers designed for the experiments are shown in Table 1.

### 2.5. siRNA Transfection

The cells reaching 50% confluence were seeded and inoculated (24 h) in a solution of siRNA sequences and Lipofectamine 2000 reagent (Gibco-BRL, Burlington, ON, Canada). ST2 siRNA, Ro60 siRNA, and SOX17 siRNA sequences were all presented as sense and antisense. qRT-PCR presented an efficiency above 80% after testing the target genes in total RNA extractions.

### 2.6. Western Blotting (WB)

Proteins separated from cell lysates were treated with electrophoresis isolation before membrane transfer. The specimens were then processed for 2 *μ*g/ml rabbit anti-Ro60 IgG (Abcam) incubation. Likewise, IL-33 (cat. no. ab187060), ST2, SOX17 (cat. no. ab224637), rabbit anti-nuclear factor-kappa B (NF-*κ*B), NF-*κ*B p65 (cat. no. #ab16502), *β*-actin (cat. no. ab8227), Akt (cat. no.), p-Akt (cat. no.) IgG, phosphatidylinositide 3-kinase (PI3K; cat. no.), p-PI3K (cat. no.) (Abcam), and CCL2 or ICAM-1, all with the concentration of 2 *μ*g/ml, served as *Ι* antibodies. Subsequently, 2 *μ*g/ml biotinylated goat anti-rabbit IgG (cat. no. #A0277; Beyotime), a II antibody, was added. Horseradish peroxidase-streptavidin and ECL kit, both from Thermo Scientific, Waltham, MA, USA, were responsible for color development and visualization, respectively, while the densitometry quantitation was achieved with the ImageJ software (ImageJ, Bethesda, MD). Each protein's relative expression was normalized with *β*-actin.

### 2.7. Flow Cytometry (FCM)

Phycoerythrin-coupled IgG against UVB (Santa Cruz) was used for FCM. Briefly, cells were digested in 0.25% trypsin-EDTA solution. Then, they were collected and blocked in 2% BSA (1 h, 4°C), followed by two PBS rinses and 2 h of *Ι* antibody incubation (2 *μ*g/ml) at 4°C. After that, a LSRII instrument (BD Biosciences, SJO, CA, USA) was used for analysis of the stained cells after rinsing. For cell apoptosis determination, FCM was also used. In brief, the cells subjected to indicated treatments were collected and used for the preparation of cell suspension in annexin binding buffer, followed by the addition of 7-aminoactinomycin D (BD Biosciences) and annexin-phycoerythrin. The prepared mixture was analyzed immediately by FCM. Data processing was made by FlowJo 7.6.1, software provided by Tree Star, Ashland, OR, USA.

### 2.8. Immunofluorescence (IF)

The cells, seeded on a petri plate (MatTek, MA, USA), were intervened by fluorescein isothiocyanate-labeled antibodies against Ro60 and ST2 (2 *μ*g/ml; Santa Cruz). Next, they were washed and examined using a fluorescent microscope (DFC300 FX; Leica Co., Wetzlar, Germany). For the IF of IL-33 and ST2 in frozen sections, primary antibodies against Ro60 IgG (2 *μ*g/ml) and ST2 IgG (2 *μ*g/ml) were used. 2 *μ*g/ml Alexa 594- and Alexa 488-conjugated donkey anti-goat IgG, with the cat. nos. of #ab150076 and #ab150129, respectively, were the II antibodies (2 *μ*g/ml; Abcam) used. Counterstaining of the sections was performed using the 4′,6-diamidino-2-phenylindole, and the examination was performed using the fluorescence microscope.

### 2.9. Macrophage (MP) Chemotaxis

For the determination of MP chemotaxis in vitro, the KCs were cultivated in the wells of 6-well plates and intervened by IL-33 or 10 *μ*g/ml BSA for 48 h. J774.2 MPs were supplied by the European Collection of Authenticated Cell Cultures. The J774.2 clone of MPs (1 × 10^6^/ml) was planted into an internal insert (Corning Inc., Corning, NY, USA) with an 8 *μ*m pore membrane to favor the migration of MPs. The internal insert was placed in a 0.4 *μ*m pore-size external one to particularly impede the penetration of IL-33 while allowing the penetration of nutrients in the medium and most other cytokines. After incubation of the Transwell system (2 h, 37°C), cell counting was performed on crystal violet-positive cells that remained on the membrane surface.

### 2.10. IFN-*α* Stimulation

An Enzyme-Linked Immunosorbent Assay (ELISA) was conducted to examine IFN-*α* concentration in the supernatant of J774.2 MPs seeded alone in culture dishes. Next, KCs were immersed in a culture medium with the addition of the recombinant mouse IFN-*α* (7 pg/ml; PBL Assay Science, Piscataway, NJ, USA) or J774.2 supernatant with the same concentration. The cells were then lysed for protein and mRNA isolation.

### 2.11. ELISA

The concentration of Ro60 in the supernatant of KCs was determined using the Anti-SSA (Ro-60) ELISA Kit (DEIA103J) from Creative Diagnostics. In addition, IFN-*α* level in the J774.2 supernatant was determined by using the Mouse IFN-alpha ELISA Kit 42120-1 (R&D Systems).

### 2.12. Statistical Processing

GraphPad Prism performed data statistical analysis. All data were denoted by means ± SD, whose comparison between groups employed one-way ANOVA and Turkey's post hoc test. Two-tailed Student's *t*-test analyzed the difference between groups. Significance was present when *p* < 0.05.

## 3. Results

### 3.1. Upregulated IL-33 and ST2 in Cutaneous Lesions of LE Patients

**Figure 1:**
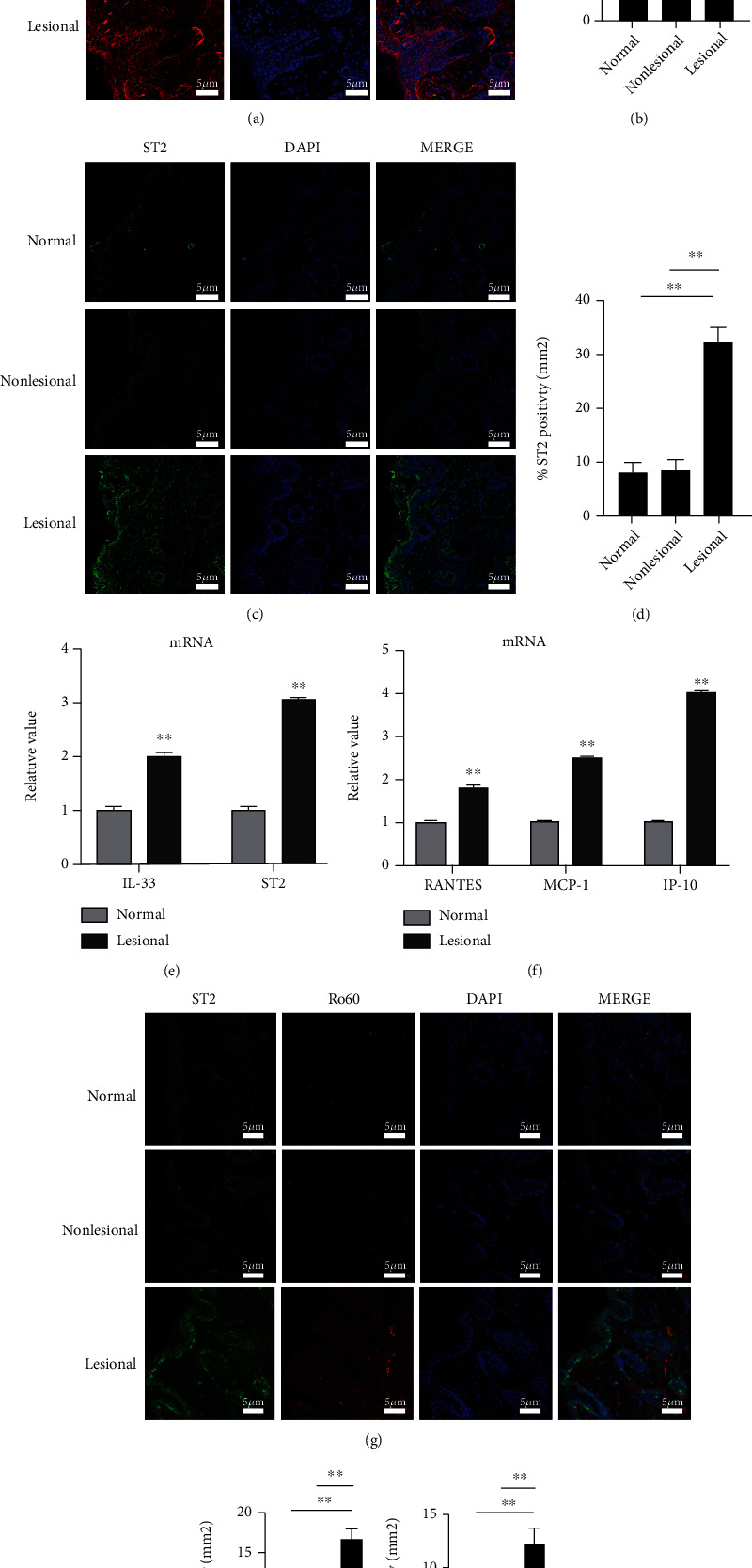
IL-33 and ST2 are upregulated in CLE patients' cutaneous lesions. (a) Immunofluorescence analysis of IL-33 in paraffin sections. (b) Numerical quantification of the positivity percentage per square millimeter of IL-33-stained sections. (c) Immunofluorescence analysis of ST2 in paraffin sections. (d) Numerical quantification of the positivity percentage per square millimeter of ST2-stained sections. (e) qRT-PCR analysis of IL-33 and ST2 mRNA expression in fresh skin tissues. (f) qRT-PCR analysis of RANTES, MCP-1, and IP-10 mRNA in fresh skin tissues. (g, h) Immunofluorescence analysis of Ro60 colocalization and ST2 expression in lesional skin tissues. Normal samples (*n* = 10), nonlesional samples (*n* = 15), and lesional samples (*n* = 15). Data points and error bars represent mean ± SEM. Representative images are shown. Bar = 50 *μ*m. ^∗^*p* < 0.05, ^∗∗^*p* < 0.01. CLE: cutaneous lupus erythematosus.

IF was utilized to measure IL-33 and ST2 contents in lesional, nonlesional, and normal cutaneous biopsies. Significantly upregulated IL-33 was found in lesional skin compared with normal controls and nonlesional skin (Figures 1(a) and 1(b)). Similarly, the IF intensity of ST2 in the lesional sections was significantly upregulated versus normal or nonlesional tissues (Figures 1(c) and 1(d)). Furthermore, the detection of mRNA expression by qRT-PCR indicated increased IL-33 and ST2 mRNA in lesional skin compared with the control samples (Figure 1(e)). Moreover, we observed upregulated P-10, RANTES, and MCP-1 mRNA levels in lesional specimens (Figure 1(f)). Confocal microscopy analysis of ST2 and Ro60 expression indicated increased expression of these proteins in the lesional tissues compared with normal specimens, and there was an overlap or colocalization of these proteins (Figures 1(g) and 1(h)).

### 3.2. IL-33 Enhances the Effect of UVB on Gene Expression in KCs

**Figure 2:**
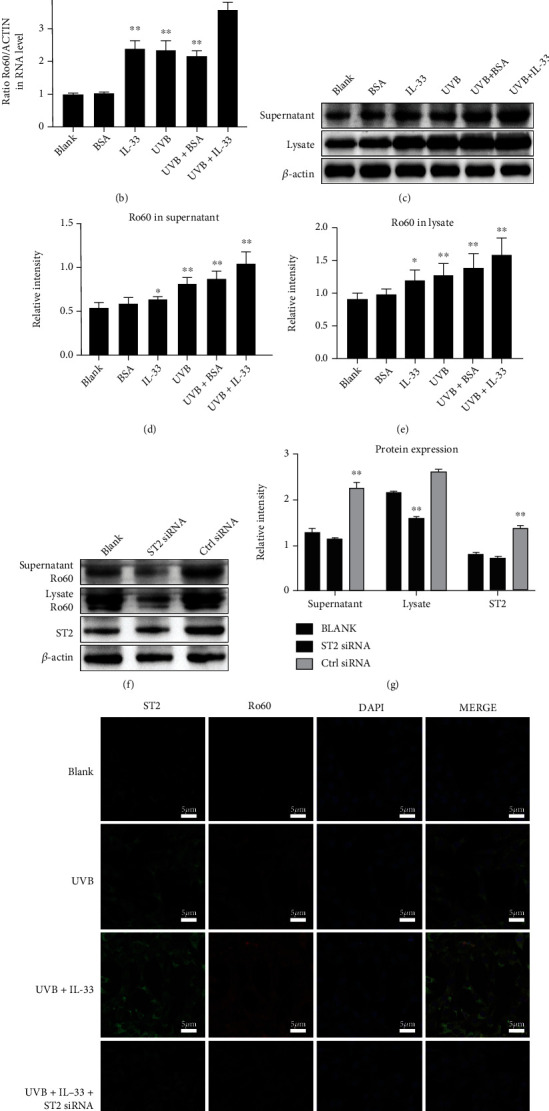
IL-33 promotes proinflammatory cytokine secretion and Ro60 expression in murine KCs upon UVB irradiation. *In vitro*, the PAM212 KCs were subjected to UVB irradiation, either alone or combined with IL-33 and bovine serum albumin (BSA) stimulation. (a) qRT-PCR determination of ST2, SOX17, CCL2, RANTES, ICAM-1, MCP-1, and IP-10 mRNA expression in KCs. (b) qRT-PCR detection of Ro60 mRNA in KCs. (c) Western blotting detection of Ro60 protein in cell lysates and supernatants of KCs. (d) Densitometry analysis of Western blotting bands of supernatant Ro60. (e) Densitometry analysis of Western blotting bands of cell lysate Ro60. (f) Western blotting analysis of Ro60 in cells transfected with ST2 siRNA or control siRNA and subjected to UVB and IL-33 treatments. (g) Densitometry analysis of Western blotting bands of Ro60 in ST2 siRNA- or control siRNA-transfected cells with UVB and IL-33 treatments. (h, i) Immunofluorescence analysis of ST2 and Ro60 in KCs subjected to different treatments. Data were obtained from three experiments conducted independently. Data points and error bars denote mean ± SEM. Bar = 5 *μ*m. ^∗^*p* < 0.05, ^∗∗^*p* < 0.01, and ^∗∗∗^*p* < 0.001. KC: keratinocyte.

To investigate the role played by IL-33/ST2 activation in gene expression, KCs were treated with UVB irradiation, either alone or combined with UVB+IL-33 stimulation. qRT-PCR results identified markedly elevated MCP-1, IP-10, RANTES, and ST2 mRNA levels after UVB irradiation (Figure 2(a)). Furthermore, we found that the combination of UBV irradiation and IL-33 stimulation further enhanced the effect of UVB on the expression of MCP-1, IP-10, RANTES, and ST2 (Figure 2(a)). Additionally, UVB increased the mRNA expression of Ro60 in KCs, and this effect was further promoted by IL-33 stimulation (Figure 2(b)). Similar results of the expression of Ro60 at protein level were determined by WB in both the cell lysates and culture supernatants (Figures 2(c)–2(e)). Moreover, the silencing of ST2 by ST2 siRNA abrogated the effects of IL-33 on Ro60 expression in UVB-irradiated KCs, as indicated by WB results (Figures 2(f) and 2(g)). Interestingly, IF results further confirmed the effect of ST2 siRNA on Ro60 expression in KCs treated with UVB+IL-33 (Figures 2(h) and 2(i)).

### 3.3. NF-*κ*B and PI3K/Akt Axis Mediates IL-33 to Enhance Ro60 Expression

**Figure 3:**
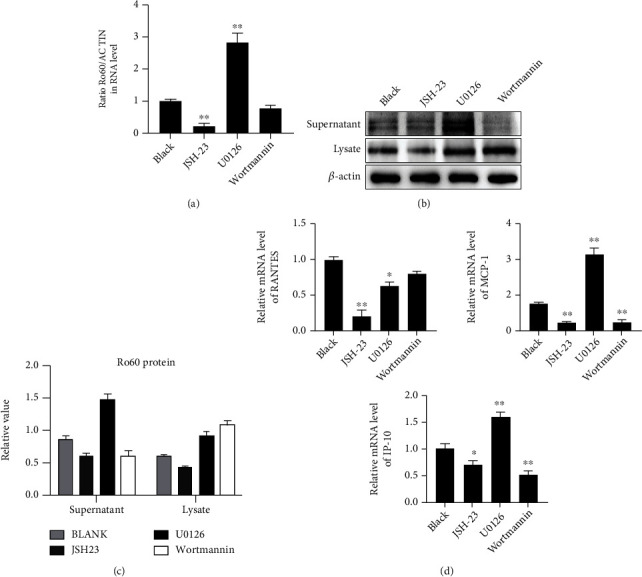
Inhibition of the PI3K/Akt and NF-*κ*B Axis affects the regulation of Ro60 and proinflammatory cytokines by IL-33/ST2 in KCs. PAM212 cells cultured in vitro were treated with ultraviolet B irradiation and IL-33 stimulation. (a) qRT-PCR determination of Ro60 mRNA expression in different treatment groups. (b) Western blotting determination of Ro60 protein expression in supernatants and cell lysates in different treatment groups. (c) Densitometry analysis of Western blot bands. (d) qRT-PCR detection of RANTES, MCP-1, and IP-10 mRNA levels in KCs. Data were obtained from three experiments conducted independently. Data points and error bars denote mean ± SEM. ^∗^*p* < 0.05, ^∗∗^*p* < 0.01. KC: keratinocyte.

To explore the mechanism of IL-33/ST2 activation of Ro60 expression, KCs received UVB and IL-33 interventions after pretreatment with JSH-23, U0126, and WM. The mRNA expression of Ro60 was increased by the treatment with U0126 was significantly higher than that in JSH-23 and WM treatments (Figure 3(a)). Additionally, protein expression levels detected by WB showed a similar tendency in KCs after pretreatment with the above inhibitors (Figures 3(b) and 3(c)). Moreover, IP-10, RANTES, and MCP-1 mRNA levels were decreased by WM and JSH-23 but increased by U0126 (Figure 3(d)).

### 3.4. IL-33/ST2 Activation Induces MP Chemoattraction in KCs

**Figure 4:**
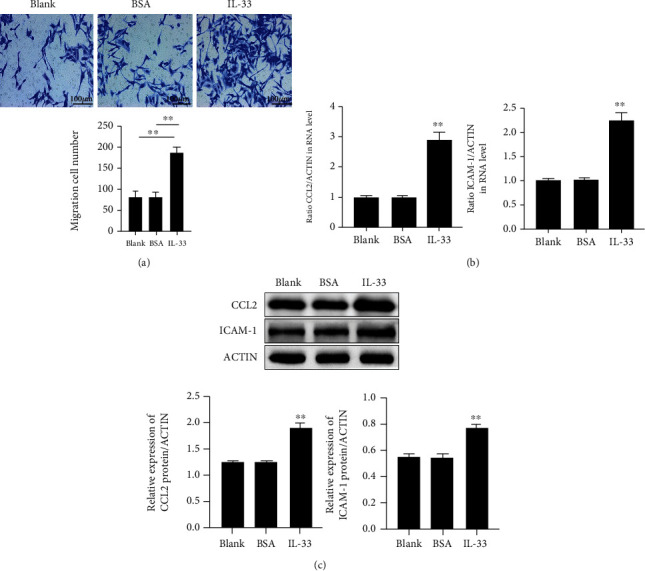
Macrophage chemoattraction is induced by IL-33/ST2 activation in KCs. (a) Transwell detection of the migration of macrophages in different treatment groups and numerical representation. (b) Detection of ICAM-1 and CCL22 mRNA by qRT-PCR in IL-33-stimulated KCs. (c) Western blot analysis of ICAM-1 and CCL22 expression levels in the supernatants collected from IL-33-stimulated KCs. Data were obtained from three experiments conducted independently. Data points and error bars denote mean ± SEM. ^∗^*p* < 0.05, ^∗∗^*p* < 0.01. KC: keratinocyte.

To study the influence of IL-33/ST2 on MP chemostasis, we performed Transwell coculture assay. It was found that the treatment of KCs with IL-33 promoted the migration of MPs (Figure 4(a)) and upregulated the mRNA (Figure 4(b)) and protein (Figure 4(c)) expression levels of ICAM-1 and CCL2.

### 3.5. SOX17 Is Critical in the IL-33 Effect on KCs

**Figure 5:**
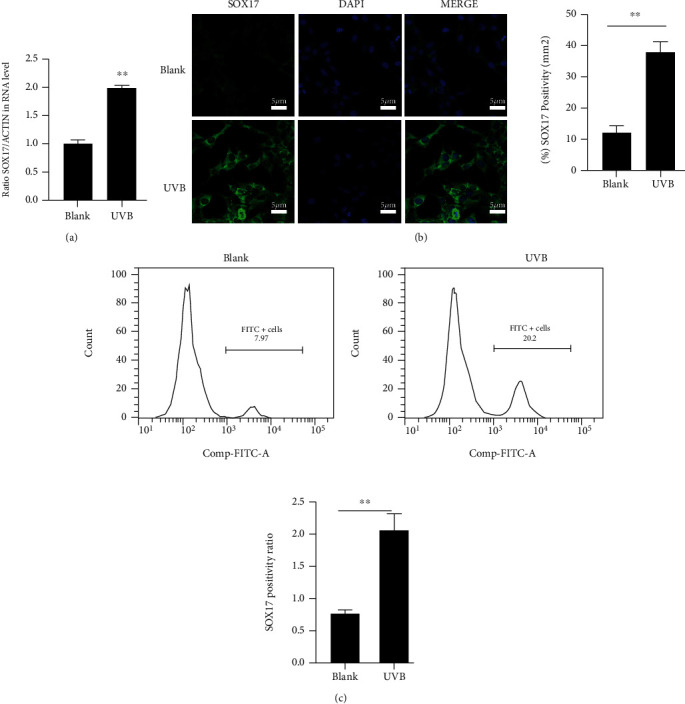
Ultraviolet B (UVB) irradiation enhances SOX17 expression in murine KCs post-UVB irradiation. (a) qRT-PCR determination of SOX17 mRNA expression in KCs following UVB irradiation. (b) Immunofluorescence analysis of SOX17 expression in KCs following UVB irradiation. (c) FCM detection of SOX17 expression in KCs. Data were obtained from three experiments run independently. Data points and error bars denote mean ± SEM. ^∗∗^*p* < 0.01. KC: keratinocyte.

**Figure 6:**
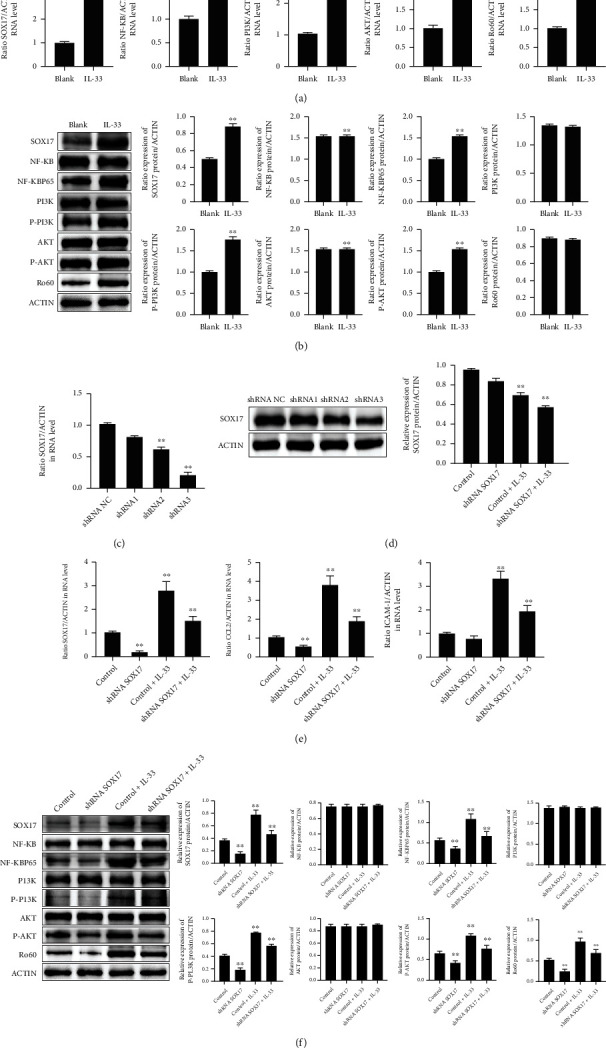
SOX17 is pivotal in IL-33's effect on KCs. (a) Detection of SOX17, NF-*κ*B, PI3K, AKT, and Ro60 mRNA expression in KCs following IL-33 treatment by qRT-PCR. (b) Western blot determination of SOX17, NF-*κ*B, NF-KBP65, PI3K, p-PI3K AKT, p-AKT, and Ro60 expression in KCs following IL-33 irradiation. (c) qRT-PCR determination of SOX17 expression in KCs after SOx17 siRNA transfection. (d) Western blot determination of SOX17 expression in KCs after SOX17 siRNA transfection. (e) qRT-PCR determination of SOX17, CCL2, and ICAM-1 mRNA expression in KCs following IL-33 stimulation. (f) Western blot determination of SOX17, NF-*κ*B, NF-KBP65, PI3K, p-PI3K AKT, p-AKT, and Ro60 expression in KCs following SOX17 siRNA transfection and IL-33 treatment. Data were obtained from three experiments conducted independently. Data points and error bars denote mean ± SEM. ^∗^*p* < 0.05, ^∗∗^*p* < 0.01. KC: keratinocyte.

To investigate the role played by SOX17 in the photosensitization effect of IL-33, the level of SOX17 in KCs irradiated with UVB was quantified by RT-qPCR. SOX17 mRNA expression was found to be increased by UVB irradiation (Figure 5(a)). Moreover, IF (Figure 5(b)) and FCM (Figure 5(c)) further confirmed the effect of UVB on the expression of SOX17 in the KCs. In the KCs, IL-33 increased the mRNA levels of SOX17, NF-*κ*B, PI3K, and Ro60 (Figure 6(a)). Moreover, IL-33 upregulated the protein expression of SOX17, NF-KBP65, P-PI3K, P-AKT, and Ro60 (Figure 6(b)). To explore the impact of SOX17 on Ro60 expression, we further transfected KCs with the SOX17 siRNA. SOX17 mRNA and protein levels were observed to be statistically inhibited by SOX17 siRNAs (Figures 6(c) and 6(d)). Furthermore, the transfection of the most efficient SOX17 siRNA markedly lowered SOX17, CLL2, and ICAM-1 mRNA expression, no matter with or without IL-33 stimulation (Figure 6(e)). Furthermore, WB analysis indicated that the inhibition of SOX17 was followed by decreased expression levels of SOX17, NF-KBP65, P-PI3K, P-AKT, and Ro60, independent of IL-33 stimulation (Figure 6(f)).

## 4. Discussion

This study found that the IL-33/ST2 axis was activated in CLE patients' cutaneous lesions. Stimulation of KCs with IL-33 improved the effect of UVB on regulating Ro60 and proinflammatory cytokines via the PI3K/Akt and NF-*κ*B pathways while markedly enhancing MP chemostasis. Besides, UVB irradiation upregulated SOX17 in KCs while ST2 or SOX17 inhibition inhibited the effect of UVB irradiation on Ro60 expression. Thus, we suggest that IL-33/ST2 axis activation promotes Ro60-regulated photosensitization in KCs via SOX17 activation.

Literature has indicated that IL-33/ST2 is involved in the nosogenesis of a wide range of skin diseases, including atopic dermatitis and psoriasis [35–37]. However, its role in CLE remains to be clarified. The regulatory relationship between IL-33 and UVB irradiation has been reported in various skin diseases. But the relationship between UVB irradiation and ST2 needs further exploration. Hence, this research is the first of its kind to reveal the status of IL-33/ST2 signaling activation in CLE and its promotional role in the photosensitization of KCs. These results suggested that the IL-33/ST2 axis constitutes a feasible target for the management of CLE and other luputic diseases. Similar trends were found between the expression of RANTES, MCP-1, IP-10, and Ro60 and IL-33/ST2 signaling activation; moreover, the silencing of ST2 indicated the regulation of RANTES, MCP-1, IP-10, and Ro60 by the IL-33/ST2 axis, suggesting that the IL-33/ST2 pathway is critical in the regulation of Ro60-mediated downstream inflammatory markers and autoimmunity. RANTES, IP-10, and MCP-1 have been reported to be upregulated in skin-related disease. The role of Ro60 in the pathogenesis of CLE and other skin-related diseases has also been reported, suggesting that the processes regulated by Ro60 and these inflammatory markers may be potential therapeutic targets for CLE and that IL-33/ST2 may be the direct and efficient target.

UVB is the main cause of photosensitivity in CLE and affects a number of factors including Ro-related proteins. For instance, it was reported that Ro52 regulates photosensitization via regulating the NF-*κ*B and TRAF2/TNFR-related signaling pathways. There are also studies revealing the role of Ro60 in the photosensitization of KCs in LSE and other skin diseases, but no research has been conducted before on the underlying mechanism. Herein, Ro60 was upregulated in the cutaneous lesions of CLE and its expression was promoted by the activation of IL-33/ST2 under UVB irradiation.

Studies have indicated the regulatory relationship between IL-33 and NF-*κ*B [38–40]. In this research, we found that the IL-33-induced Ro60 activation in KCs could be restrained by PI3K/Akt or NF-*κ*B inhibition but upregulated after the inhibition of MAPK/ERK, which indicated that NF-*κ*B and PI3K/Akt signaling pathways mediate the regulation of Ro60 by IL-33. In addition, it suggests that these pathways participate in the ameliorating effect of IL-33/ST2 on KC photosensitivity. IL-33 has been indicated to influence SOX17 via activating CCL2. Thus, SOX17 may be involved in the role played by IL-33 in KCs via regulating SOX17. In this study, UVB-irradiated or IL-33-stimulated KCs expressed higher mRNA levels of SOX17. In addition, the transfection of SOX17 siRNA downregulated Ro60 protein expression in KCs treated with UVB irradiation and IL-33 stimulation. Moreover, silencing SOX17 affected the expression of inflammatory markers. Hence, the ST2-SOX17 axis may play a part in the IL-33/ST2-regulated Ro60 expression and inflammatory pathways. However, there are some limitations that still need to be addressed in this study. Although Ro60 is found to be upregulated in the cutaneous lesions of CLE and its expression is promoted by the activation of IL-33/ST2 under UVB irradiation and the ST2-SOX17 axis may be involved in the process, the exact mechanism beneath this regulation needs to be clarified. Besides, extra environmental factors and the interaction between IL-22 and STE in CLE are worthy of further exploration.

## 5. Conclusion

Conclusively, IL-33/ST2 is activated in CLE and enhances the effect of UVB on Ro60 expression in KCs. Additionally, IL-33/ST2 activation in KCs induces MP chemostasis, which indirectly may lead to elevated Ro60 expression in cells. Moreover, UVB irradiation significantly induces SOX17 expression in KCs. SOX17, as well as NF-*κ*B and PI3K/Akt axes, is involved in IL-33-upregulated Ro60 expression. In CLE, targeting the IL-33/ST2 signaling may be a novel strategy in managing Ro60-regulated photosensitivity.

## Figures and Tables

**Table 1:** Primer sequences.

	Forward primer (5′ → 3′)	Reverse primer (5′ → 3′)
IL-33	AGACAGGAAAGCTGATGCCC	CTGCAAAATACCCCAACACCC
ST2	TGCCTGAAGTGCTATGTGGG	ATGGAGACATAGGCCTTTCAAGT
RANTES	TAACCTTCTGCCCTGGGCTT	GGCAACTGATGCTTCCCAAC
MCP-1=CCL2	ATGGACCATCCAAGCAGACG	CCCTTGCTCCACAAGGAAGA
IP-10	TCTGACTCCCAAGATTGCCG	ACTGTGCTAACCTTCTCTGCTG
*β*-Actin	TGGTGAGCTGCGAGAATAGC	TCCGACCAGTGTTTGCCTTT
ICAM-1	TCCTCACCGCCTGTTGTATC	ACTTCCCCTCTCATCAGGCT
Ro60	TTTGCTGGAGGTGTCCATCC	TCTATAACGGACACTGGCTCC
NF-*κ*B	TTCCCGATCTGAGTCCAGGT	GCTTGTCTCGGGTTTCTGGA
PI3K	TTGAGAGCAGCAGCCAATCA	TGTTGTCTGTTGCCAAGGGT
Akt	CAGCCGTTGGACAAATCACC	GGAGGTTTTTGGGCTTGCG
Sox17	CCCAAAACCAGGGGTGTGTA	TTTTGGTCTCCTGGAAGCGG

## Data Availability

The labeled dataset used to support the findings of this study are available from the corresponding author upon request.
